# Book Review

**Published:** 1901-06

**Authors:** 


**A Text-Book of Histology, including Microscopic Technic.** By A. A. Böhm, M.D., and M. von Davidoff, M.D. Edited by G. Carl Huber, M.D. Authorised Translation from the Second Revised German Edition, by Herbert H. Cushing, M.D. Pp. 501. London and Philadelphia: W. B. Saunders & Co. 1900.—This is a translation of a well-known German text-book into the American language, and the title of an English paper that has been added to the bibliography had to be Americanised before it could be included in this work; but even after making full allowance for differences of idiom, the translation leaves something to be desired. Another sign of its American origin is shown by the fact that the editor only vaguely indicates in the preface those places in which "additions to the German text have been freely made"; but perhaps we are unduly sensitive about such little things on this side of the water. Dr. Huber, however, has added a large number of illustrations to the book, most of which were much needed. These he has not omitted to acknowledge. The most valuable additions to the text are those concerning the innervation of various organs and tissues, a branch of histology to which Dr. Huber has paid much attention. There has been little attempt to adapt the book to the use of English teachers of physiology, and we are afraid that this edition will do little to popularise Böhm and Davidoff as an English text-book. Among instances of loose translation, others of which have been noticed, we may mention that of the word "Verbreitung," p. 269 (*Technic*, 224), which is rendered "distribution." The word "demonstration" would have been correct, and would have made sense, which "distribution" does not. Again, on p. 73 (*Technic*, 125) the word "demonstration" should have been used, not "formation," in translating "Darstellung." The word "formation" completely alters the sense.

**Manual of Physiology.** By G. N. Stewart, M.D. Fourth Edition. Pp. 894. London: Baillière, Tindall and Cox. 1900.—The first edition of this text-book of physiology appeared in 1895, and already it has become one of the most popular. It covers all that is needed for any English examination, and is brought out at a reasonable price—two not unimportant points when a student is selecting his text-book. But its popularity rests also on other grounds: it is written in a very readable style and in a manner that appeals to the memory; moreover, the author does not assume that the student has his physics and chemistry at his fingers' ends, and frequently digresses to give a *resumé* that does not strictly belong to physiology, but which is essential to comprehension of the subject. The chief additions to this edition are a table for testing solutions of the more common proteids and carbo-hydrates, also those to the section on the nervous system, and generally the book has been brought up to date. We have noticed a few slight errors that need correction in the next edition. Several of the page references in the practical exercises on the first chapter are incorrect: the proportions of the ingredients in A.C.E. mixture are given as equal parts, and the fractions for the conversion of Fahrenheit and centigrade scales are transposed in the appendix.

**Golden Rules of Physiology.** By I. Walker Hall, M.B., and J. Acworth Menzies, M.B. Pp. 80. Bristol: John Wright & Co. [1900.]—We are quite at a loss to understand why such books as this are written. Perhaps it is an attempt to imitate the modern system of medication by means of tabloids, tablets, *et hoc genus omne*. But just as some pills become exceedingly hard when compressed, so the elements of a science become indigestible when too much condensed; and statements which, were space allowed for explanation, would appear correct, can only result in producing an altogether misleading impression when sent naked into the world. The book before us abounds with such statements, and our sincere advice to every student is—"*Avoid it, at all events until you have passed your examination.*"

**On Some Cirrhoses of the Liver.** By William Butler Cheadle, M.D. Pp. viii., 109. London: Smith, Elder & Co. 1900.—This book, which comprises the Lumleian Lectures for 1900, expresses the views of an observant physician of experience upon the varieties of cirrhosis. A remark is made which many of us no doubt may have found to be true. He states: "It is surprising how a definite observation, stated with authority, holds sway, and its general application remains unquestioned, as all-comprehensive, without further examination or enquiry." As an illustration of this statement, he instances the influence exerted by the opinions of Laennec, and the description of a variety of cirrhosis by Hanot. Dr. Cheadle disbelieves, and all pathologists of experience no doubt now would agree with him, that the atrophic form of cirrhosis never follows the larger variety. He also thinks that Hanot's cirrhosis, if it exists at all, must be very rare. He has never met with a case, although he has looked carefully for an example for sixteen years. In the comments on the possibility of hypertrophic cirrhosis contracting down into the atrophic form, no reference is made to Graham Steell's suggestion that an enlarged liver due to congestion, associated with alcoholic cardiac failure, may not uncommonly be mistaken for a large cirrhotic liver. Such livers Dr. Steell has seen diminish in size as the condition of the heart improved under rest in hospital. The common association of a large fatty liver with alcoholism appears to have been overlooked. Its presence has a bearing upon several points referred to, of greater importance than that to which reference has just been made. Small though the book is, the numerous features of interest of which it speaks cannot be touched upon in a short review. Such facts as these, however, may be worthy of mention: in eight cases of jaundice, associated with cirrhosis, four were in the atrophic form, four in the hypertrophic. Dr. Cheadle has met with two cases of cirrhosis apparently due to obstruction of the common bile duct by gallstones, a pathological sequence for a long time considered doubtful in the human being. Of eight cases of cirrhosis in children, in two there was a history of congenital syphilis, in two of alcoholism, and in four no cause could be made out. Other points, clinically more important, such as the question of prognosis and of treatment, we must leave our readers to find for themselves in the lectures.

**The Johns Hopkins Hospital Reports.** Vol. VIII., Nos. 3—9. Baltimore: The Johns Hopkins Press. 1900.—The high character of the work associated with the Johns Hopkins Hospital is fully maintained in the present volume of reports, which deals exclusively with enteric fever in its medical, surgical, and bacteriological aspects. Space will only permit of notice of a few of the articles contained in the volume, but all give evidence of laborious research, and will well repay study. Thayer's "Observations on the Blood in Typhoid Fever" will long be regarded as a standard for reference. Of especial interest is his observation that by carefully watching the leucocyte count it is possible to diagnose a pre-perforative stage of intestinal ulceration; and in further articles both Finney and Cushing confirm this observation, and urge that this is the period at which operative procedures may be undertaken with the greatest prospect of success. We note that Thayer still retains the objectionable term "*neutrophile*" leucocyte. Young's report of a case of chronic cystitis of seven years' duration due to the typhoid bacillus is clear and convincing, but from Dobbin's report on a case of puerperal infection with the bacillus typhosus it does not seem to us at all clear that this organism played any part in the causation of the disease. Osler's analysis and general summary of the 829 cases of enteric fever admitted to the hospital during the period 1889—1897, is a most valuable addition to the bibliography of the disease, and one which will constantly be consulted by clinicians in the future. In reading through the volume, one cannot fail to be struck with the freshness of methods of research and the originality of thought that marks throughout the work of the various authors.

**Transactions of the Association of American Physicians.** Vol. XV. Philadelphia: Printed for the Association. 1900.—Of the many interesting papers here collected, that by Dr. Trudeau, of Saranac Lake, N.Y., on the Sanitarium treatment of incipient pulmonary tuberculosis, is of especial use at the present time, inasmuch as it gives the results which have been obtained in the Adirondack Cottage Sanitarium, one of the pioneer institutions of America, both by the ordinary method and by the exhibition of tuberculin. The statistics do not give any very decided difference between a series of 50 treated by tuberculin and those not so treated, and Dr. Trudeau remarks that "the immunising effect of tuberculin as observed in the human subject would appear to be more uncertain both as to degree and duration, and immunity is still the unattained goal of experimental research." The Sanitarium treatment gives a general result of 72 per cent. discharged apparently cured.

**Transactions of the Royal Academy of Medicine in Ireland.** Vol. XVIII. Dublin: Fannin and Co., Ltd. 1900.—It is impossible to review adequately such a volume as this, which contains within itself the collected work of the leaders in Ireland of the various branches of medicine, surgery, obstetrics, pathology, state medicine, anatomy and physiology. There are two papers on "Epidemic Cerebro-Spinal Meningitis," a subject to which we have given some attention of late. "The disease was first recognised in Ireland in 1846. Again, in the autumn and winter of 1866-67 a very considerable outbreak occurred here, and also in 1885-86 a minor epidemic appeared . . . . . It appears that we have entered on another epidemic of this disease in Dublin." Numerous cases are reported shewing the characteristic features of the disease, its bacteriology, and the use of the lumbar puncture and Kernig's sign in diagnosis.

**The Medical Annual.** Bristol: John Wright and Co. 1901.—This compendium of everything useful, with a minimum of literary padding and a maximum of pages of the advertisement type, is in its nineteenth year. During this period it has had many and cosmopolitan contributors, but the list varies much from year to year, and the publishers have shown a wise selection in their choice. Part I., "The Dictionary of New Remedies," is, as before, by Dr. William Murrell, and it contains the latest information on antitoxins and the various forms of serum. The same author contributes a paper on "The Light Treatment in Lupus and Allied Conditions. Part II., "The Dictionary of New Treatment," the major part of the volume, consists of 506 pages by many authors, one of the most distinguished being Dr. H. P. Loomis, of New York, whose article on "Phthisis" commences with the remark that "the salient feature of the year in pulmonary tuberculosis might be said to be the interest evinced everywhere in the open-air treatment for the patient as distinct from the serum treatment or germicidal attempt upon the bacillus, the building up and strengthening of the fortress by means of Nature's remedies rather than the arrest of the invading foe by drugs and antitoxins." An article by Professor Charles Ruata, of Perugia, on "Tuberculosis," advocates the constant inhalations of antiseptic volatile substances—"the compounds of *creasote*, mixed with *volatile oils* and *chloroform*, or *formalin*, conveniently diluted with alcohol and a little chloroform to make it bearable, give excellent results," whereas he maintains that remedies given by the mouth are useless, and that no serum has been found to have the least power. Numerous articles comprised under the heading of "Ophthalmology" are of local origin, having been written by Mr. F. Richardson Cross. Part III., "Miscellaneous," contains much useful information on sanitary science, legal decisions affecting medical men and public health, a review of new inventions, and pharmaceutical and dietetic articles, books of the year, lunatic and idiot asylums, homes for inebriates in Great Britain and Ireland, Invalids' Homes, nursing institutions and associations, and various directories, including an index to life assurance offices. Clearly the publishers have succeeded in making the book useful to every medical man, who should keep it constantly within reach for consultation in all difficulties.

